# Atlanta Academy of Medicine

**Published:** 1876-11

**Authors:** R. W. Westmoreland

**Affiliations:** Reporter


### Reports of Societies.

### ATLANTA ACADEMY OF MEDICINE.

R. W. WESTMORELAND, M.D., Reporter.

Atlanta, October 3, 1876.

Meeting called to order by the President, Dr. Miller.

Dr. Calhoun reported a very interesting case of foreign body in the eye. The length of time it remained without causing more trouble was something very unusual. The patient, from Carroll county, Georgia, some three months ago, while hammering on a plow, was struck in the left eye by a spicula three-quarters of an inch long, the iron entering at the inner corneo-sclerotic junction, and ranging diagonally passed through the ball and rested half an inch of its extent along the external side of the optic nerve; one-quarter of an inch projected into the ball. Considerable pain and inflammation came on, but soon subsided, and, with the exception of loss of vision in that eye, no further difficulty was experienced until six weeks afterwards. At this time the pain was renewed, and the right eye became involved sympathetically. Upon examination with the ophthalmoscope, there was discovered what appeared to be a tumor growing into the vitreous humour from the posterior part of the iris. This, however, proved to be an extensive effusion, the result of the injury. The operation of enucleation was performed, and not until the instrument came in contact with the body was its situation discovered. The fact that it remained so long in this situation, rasping the optic and ciliary nerves, without arousing an immediate and more serious trouble, is very exceptional and unusual.

Dr. John M. Johnson read a paper on puerperal eclampsia, in which he advocated very decidedly the practice of venisection, and desired expression of opinion by the Academy.

Dr. J. G. Westmoreland formerly believed that the congestion of the brain, caused by the mechanical influence of labor-pains, resulted in convulsions, and bled to relieve this condition. Cited a case where this practice was resorted to, and three or four bleedings had without benefit, when, upon the administration of chloroform, the convulsions ceased. Spoke of a scarcity of urine for a day or two before the attack being a prominent symptom. Albumen is generally found in the urine under these circumstances, which speedily disappears under the use of chloroform given by the stomach. He has found the same result from its use in all cases of albumenuria. His explanation of the beneficial effects of the remedy in puerperal eclampsia is that it destroys the uræmic poison resulting from inactivity of the kidneys, and at the same time gives direct excitement to the obtunded function of the brain. Did not fail to bleed in plethoric patients suffering from eclampsia.

Dr. W. F. Westmoreland alluded to the pathological difference between spurious and true eclampsia by late pathologists. Favored the opinion that true eclampsia resulted only from uræmic poisoning. Had used chloroform successfully in albumenuria under various circumstances, and favored its use in eclampsia, in twenty-drops doses, three or four times a day, as preventive to the convulsions; also bled in plethoric subjects. Mentioned that chloral, either hypodermically, by the rectum, or mouth, was spoken of very favorably by English doctors.

Dr. Miller gives chloroform to remove the condition that produces the convulsion—the albumenuria. The remedy does this, he does not know how, but supposes that it is by inducing a change of albumen into sugar, producing a temporary diabetes, which results in a cure.

Dr. John M. Johnson thought that bleeding acted by diminishing the amount of the uræmic poison, and by correcting the deranged condition of the kidneys. Gives chloroform, too, but has found that repeated doses excite nausea and disgust for the remedy. In this event he substitutes digitalis and squills.

Dr. Miller stated that in plethoric patients, where there was danger of effusion and congestion, as the result of the convulsion, he would bleed. When there was evidence of blood poisoning, he would use chloroform. Never has seen the disgust and nausea, resulting from the use of chloroform, that Dr. J. Spoke of.

Dr. Baird discussed the theory of Dr. Miller as to the change of albumen into sugar, and could not understand how it could be done.

Dr. Miller stated that it was only supposition on his part, and did not pretend to explain the theory.

---

Atlanta, Oct. 10, 1876.

Meeting called to order; the President, Dr. Miller, in the chair.

Dr. W. F. Westmoreland, alluding to the practice of heroic injections of water for the cure of obstructed bowel, expressed his belief in the fallacy of such practice. Reported a case which, from the symptoms of vomiting, excessive pain, and absence of action on the bowels, was evidently one of intussusception. Injections to the amount of one gallon and a half of water was given, manifestly without benefit, as the vomiting continued to increase, and to assume the stercoraceous character. Whereupon he changed his plan of treatment, by administering opium, and carrying its effect to the point of semi-narcotism. In addition to this, ice was freely applied to the abdomen. Thirty hours after the commencement of this treatment the patient had an action of the bowels. Referred to the case of Dr. Cumming, of Marietta, which was cured by this plan of semi-narcotism. His object is to relax and paralyze the muscular coat of the bowels, and to control the inflammation always resulting in these obstructions. Application of cold is very effective, though he does not know the manner in which it brings about the benefit. Had often used, during the war, the cold douche in colic and persistent constipation with very favorable results.

Dr. Armstrong reported the prevalence of scarlet fever in his practice, and desired to know if any member had met with the disease.

Dr. Baird discussed electricity as a therapeutical agent, and reported a case of obstinate gastralgia treated by its administration. The case was one of long standing, having come on gradually, until her suffering was most intense, resembling colic. Every remedy was tried without avail, until galvanism was applied along the right pneumogastric nerve. The next day after application she reported immense improvement. On the second application of the current she was entirely relieved, demonstrating, from the locality through which the current was directed, that the right pneumogastric nerve was the seat of the difficulty.

Dr. John M. Johnson inquired as to the prevalence of diphtheria in the city. Also called attention to the therapeutical use of hot water internally and externally, resulting in cure in two weeks. Is not certain as to the diagnosis, but the more prominent symptoms were present of Bright's disease of the kidneys. Another case of sore eyes, and one of dysentery, he relieved by the same means.

Dr. Head (visiting, post surgeon), in reply to Dr. Johnson's inquiry, remarked upon the uncertainty of diagnosis of diphtheria in general; many sore throats being pronounced diphtheretic that were not. Recollects to have seen but about two cases of true diphtheria. In these all means resorted to for cure were unavailing. Inquired of Dr. Johnson as to the diaphoresis produced by the hot water in the case of Bright's disease.

Dr. John M. Johnson answered that thorough diaphoresis and diuresis were produced.

Dr. McClellan (visiting, post surgeon) reported a case of imperforate anus in a girl fifteen years of age. A former attempt to operate had been made, but was unsuccessful. The bowels never having free action, were unusually distended. On examination a small aperture was discovered just behind the posterior commissure, through which fecal matter constantly passed, with much and constant pain. Cut through the perineum and emptied the gut, and stitched the intestine down to the incision. From some impropriety in diet, diarrhœa set up soon after, and the bowels were completely emptied, with, however, no serious result. Three weeks after, operated for ruptured perineum, and the child got well. In operating, instead of paring off the mucous membrane, he made a triangular incision, the apex of which pointed into the vagina. Bringing the parts together with cat-gut sutures, a clear surface was left, which united readily. On inquiry, replied that the sphincter was complete and normal.

Dr. Elbry (visiting, post surgeon) reported a case of internal piles, with prolapsus ani, for which he operated by ligature. Allingham's method of operating was attempted, but on account of excessive tenesmus, was abandoned, and the common mode of transfixion and ligation pursued. Vomiting, at first supposed to be the result of the ether administered, persisted to such an extent that no sustenance could be retained, while the pulse was very feeble. The patient died, and peritonitis, confined to the pelvis, was discovered.

Dr. W. F. Westmoreland inquired if there were indications of pyæmia or septicæmia, or if the peritonitis was sufficient to cause death.

Dr. Elbry thought that death occurred more from the peritonitis.

Dr. W. F. Westmoreland remarked that he imagined there might be two modes of death from the operation: 1st, from the shock; 2d, septicæmia or pyæmia.

Dr. Calhoun presented a pathological specimen taken from a man aged 45 years, who made his appearance with an enormous tumor growing from right eye. This resulted from the caustic treatment of a pterygium. When the tumor from the pterygium first showed itself, nitrate of silver was freely applied, which served to increase the growth. The tumor occupied the whole space around the eye; the lower lid was completely disorganized, and the disease extending down the cheek. He operated by sliting up the external canthus, and throwing the upper lid back, when the tumor was dissected up from the bone, and the eye-ball enucleated. Remarked that the tumor was evidently malignant in its character.

Dr. R. W. Westmoreland reported the case of an extensive burn of the left arm, implicating the elbow-joint, treated by the application of white lead paint, and cotton pads. After two weeks treatment with the carron oil, the burn not improving, but growing more painful, the lead was applied, with almost immediate cessation of pain and rapid healing of the sore. No cicatrix of any extent remained.

On motion, Academy adjourned.

